# The type III secretion system is necessary for the development of a pathogenic and endophytic interaction between *Herbaspirillum rubrisubalbicans* and Poaceae

**DOI:** 10.1186/1471-2180-12-98

**Published:** 2012-06-06

**Authors:** Maria Augusta Schmidt, Eduardo Balsanelli, Hellison Faoro, Leonardo M Cruz, Roseli Wassem, Valter A de Baura, Vinícius Weiss, Marshall G Yates, Humberto M F Madeira, Lilian Pereira-Ferrari, Maria H P Fungaro, Francine M de Paula, Luiz F P Pereira, Luiz G E Vieira, Fábio L Olivares, Fábio O Pedrosa, Emanuel M de Souza, Rose A Monteiro

**Affiliations:** 1 Department of Biochemistry and Molecular Biology, Universidade Federal do Paraná, R. Francisco H. dos Santos s/n, Curitiba, 81531-990, Brazil; 2 Department of Genetics, Universidade Federal do Paraná, R. Francisco H. dos Santos s/n, Curitiba, 81531-990, Brazil; 3 Center for Agricultural and Environmental Sciences, Pontifícia Universidade Católica do Paraná, Rua Imaculada Conceição, 1155, Curitiba, 80215-901, Brazil; 4 Department of General Biology, Universidade Estadual de Londrina, Rodovia Celso Garcia Cid PR-445 Km 380, Londrina, 86051-990, Brazil; 5 Departament of Genetics and Molecular Biology, Instituto Agronômico do Paraná, Rodovia Celso Garcia Cid Km 375, Londrina, 86047-902, Brazil; 6 Center of Bioscience and Biotechnology, Universidade Estadual do Norte Fluminense/CBB/LBCT, Av. Alberto Lamego, 2000, Campos dos Goytacazes, 28013-600, Brazil

## Abstract

**Background:**

*Herbaspirillum rubrisubalbicans* was first identified as a bacterial plant pathogen, causing the mottled stripe disease in sugarcane. *H. rubrisubalbicans* can also associate with various plants of economic interest in a non pathogenic manner.

**Results:**

A 21 kb DNA region of the *H. rubrisubalbicans* genome contains a cluster of 26 *hrp/hrc* genes encoding for the type three secretion system (T3SS) proteins. To investigate the contribution of T3SS to the plant-bacterial interaction process we generated mutant strains of *H. rubrisubalbicans* M1 carrying a Tn5 insertion in both the *hrcN* and *hrpE* genes. *H. rubrisulbalbicans hrpE* and *hrcN* mutant strains of the T3SS system failed to cause the mottled stripe disease in the sugarcane susceptible variety B-4362. These mutant strains also did not produce lesions on *Vigna unguiculata* leaves. *Oryza sativa* and *Zea mays* colonization experiments showed that mutations in *hrpE* and *hrcN* genes reduced the capacity of *H. rubrisulbalbicans* to colonize these plants, suggesting that *hrpE* and *hrcN* genes are involved in the endophytic colonization.

**Conclusions:**

Our results indicate that the T3SS of *H. rubrisubalbicans* is necessary for the development of the mottled stripe disease and endophytic colonization of rice.

## Background

*Herbaspirillum rubrisubalbicans* was originally described as the causal agent of mottled stripe disease in sugarcane (*Saccharum oficinarum*) but it can also cause red stripe disease in some varieties of sorghum (*Sorghum bicolor*) [1-5]. The mottled stripe disease was first described in Louisiana (USA) in 1932 and is characterized by the development of red streaks with white spots on the leaves of sugarcane. It is a disease of relatively small economic importance and affects sugarcane varieties B-4362 and Taiwang [3,6,7]. Inoculation with high numbers of *H. rubrisubalbicans* cells in the stems of the susceptible varieties cause typical symptoms of the disease. The point of injection becomes red and necrotic and, after seven days, red stripes are formed along the vessels near the inoculation site, accompanied by different degrees of chlorosis. At this stage the bacteria infest the protoxylem and the metaxylem of the leaves. On the twentieth day the bacteria block both xylem lumen and there is necrosis around the inoculation point [1]. The extensive bacterial colonization results in the expansion of intercellular spaces and subsequent compression of the host plant cells. Bacterial cells can eventually move from the vessels into the surrounding mesophyll, reaching the stomata and reducing the photosynthetic activity and lifetime of the leaves. Host plant responds with the production of phenolic compounds, gum, and localized cell death [1].

*H.rubrisubalbicans* can cause symptoms of red stripe disease on sorghum leaves of some cultivars after artificial inoculation. This mild disease is characterized by red stripes along the veins of the leaves near the point of inoculation, and these leaves showed dense colonization by *H. rubrisubalbicans* at 5 days after inoculation. *H. rubrisubalbicans* is restricted to the metaxylem, protoxylem and associated lacunae, which are completely filled with bacteria; this behavior is different from that observed in mottled stripe disease, where the bacteria escaped from the vascular system to the adjacent mesophyll and substomatal cavities destroying chroplasts, and revealing the mottled background [1,5].

*H. rubrisubalbicans* is also known as a PGPR (Plant Growth-Promoting Rhizobacteria). This bacterium is a component of the bacterial consortium developed by the Brazilian Agricultural Research Company (EMBRAPA) and recommended as a commercial inoculant for sugarcane [8-10].

The genes of the type three secretion system (T3SS) were first identified as hypersensitivity response and pathogenicity (*hrp*) genes in the phytopathogenic bacterium *Pseudomonas syringae* by Lindgren *et al. *[10]. Subsequent studies showed that the *hrp* genes of *P. syringae* were located in a cluster of 25 Kb. Similar gene clusters were also found in other phytopathogenic organisms [11-13]. Several hypersensitive response and pathogenicity genes of plant pathogens are homologous to genes of animal pathogens that encode components of the T3SS [14,15], and were named *hrc* (HR conserved) [16]. The T3SS is present in Gram-negative pathogens of animals and plants, and was then described in symbiotic [17], saprophytic and associative bacteria [18-20]. The T3SS consists of a secretion apparatus that delivers a series of effector proteins [21] across the inner membrane, the periplasmic space and outer membrane of bacteria into the eukaryotic cell cytoplasm. The effector proteins manipulate and control the host cell metabolism to the advantage of the pathogen and to repress defense mechanisms. Analyses of a partial genome sequence of *H. rubrisubalbicans* revealed the presence of genes homologous to the T3SS. In this work we show that *H. rubrisubalbicans* T3SS is necessary for the development of the mottled stripe disease in sugar cane and also for endophytic colonization of rice.

## Results

### Organization of the *hrp/hrc* gene cluster in *H. rubrisubalbicans* M1

**Figure 1:**
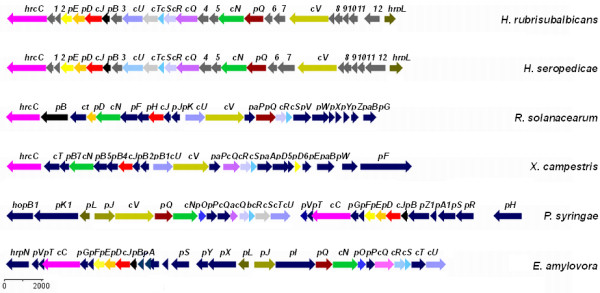
**Genetic organization of type III secretion system from *Herbaspirillum *and other phytopatogens*.*** Comparison of T3SS gene clusters from *H. rubrisubalbicans* M1 (JN256203), *H. seropedicae* SmR1 (NC014323), *Ralstonia solanacearum* CFBP 2957 (FP885907 - plasmid RCFBPv3_mp), *Xhanthomonas campestris pv campestris* (AE008922), *Pseudomonas syringae pv tomato* DC3000 (AE016853) and *Erwinia amylovora* ATCC49946 (FN666575). The *hrc* and *hrp* gene designations are sometimes replaced with *c* and *p*, respectively in *H. rubrisubalbicans* and other plant associated bacteria*.* Homologous genes are in the same color; gray genes encode hypothetical proteins found from *H. seropedicae* and *H. rubrisubalbicans*; dark blue genes encode proteins with no homology with *H. rubrisubalbicans* proteins.

The *hrp/hrc* genes cluster of *H. rubrisubalbicans* M1 contains 26 genes distributed in a 21 kb region, composed of seven *hrp*, eight *hrc*, and eleven genes encoding for hypothetical proteins (GenBank accession JN256203) (Figure 1). Based on partial homology we found that most of the genes in this cluster encode structural proteins of the T3SS, that are involved in the construction of the base and the injectiossome.

Comparison of the DNA sequence of the *hrp/hrc* cluster of *H. seropedicae* SmR1 with *H. rubrisubalbicans* showed that the genes are almost identically arranged (Figure 1). However, aminoacid sequence comparison of the proteins encoded by the *hrp/hrc* genes of both organisms showed that only five out of 26 proteins have more than 70% identity (Additional file 1: Table S1). The degree of identity between each of the deduced *H. rubrisubalbicans hrp/hrc* proteins and its counterpart from *H. seropedicae* ranged from 11% (hypothetical protein 6) to 86% (HrcS), and the respective similarity varied from 17 to 97% (Additional file 1: Table S1). The structural organization of *hrcUhrcThrcShrcRhrcQ* and *hrpBhrcJhrpDhrpE* genes of *H. rubrisubalbicans* resembles that of *H. seropedicae*, *Pseudomonas syringae*, *Erwinia amylovora*, and *Pantoea stewartii* (Figure 1). Two genes, *hrpL* and *hrpG* (JN256211)*,* which probably encode the regulatory proteins HrpL and HrpG may be responsible for the regulation of T3SS genes. In the region upstream of *hrpL* no σ^54^-dependent promoter was found, in contrast to what was observed in the *hrpL* promoter region of *Pseudomonas syringae pv. maculicola *[22,23]*.* The *hrpL* gene is located at one end of the *hrp/hrc* gene cluster while *hrpG* is located approximately 10 kb downstream from the *hrcC* gene at the other end.

**Figure 2:**
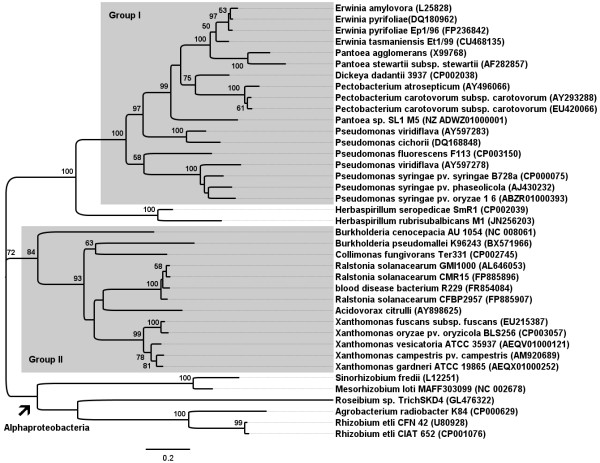
**Phylogenetic tree from *hrcN *gene sequences from Alpha and Betaproteobacteria representants.** Organisms of group I and II share similar T3SS gene cluster organization. The tree was built using the Maximum-Likelihood method with the Tamura 3-parameter model, gamma distributed rates and invariant sites (see Material and Methods for details). Bootstrap values are shown as percentage (>50%) from 1,000 replicates for each node. The tree is unrooted tree. Scale bar represents number of nucleotide substitutions per site. GenBank accession numbers are in parenthesis.

Within the Betaproteobacteria subdivision two groups of T3SS-containing organisms are observed concerning the conservation of gene order in the T3SS gene cluster members of group I include *Erwinia* sp., *Pantoea* sp., *Pectobacterium* sp., and *Pseudomonas* sp. This group includes only Gammaproteobacteria, thus far, suggesting that it is taxonomically uniform. All members of this group contain the *hrpL* gene, that encodes a sigma factor. Group II include representants of the Betaproteobacteria such as *Ralstonia* sp., *Burkholderia* sp. as well as Gammaproteobacteria, such as *Xanthomonas* sp. This group lacks *hrpL* gene but also contains HrpB or HrpX, which are transcriptional regulators of the AraC family [24]. Phylogeny of *hrcN* gene revealed that those organisms form monophyletic groups (Figure 2). Both *H. seropedicae* SmR1 and *H. rubrisubalbicans* M1 contain the *hrpL* gene and show T3SS gene organization similar to that observed in organisms of the group I. However, the phylogeny of *hrcN* gene shows that, the two *Herbaspirillum* species clustered closer but outside from members of the group I-*hrcN* cluster (Figure 2), suggesting a distant evolutionary relationship and supporting a hybrid system as suggested by Pedrosa *et al. *[25] for *H. seropedicae* SmR1, what may partially explain the differences observed in gene organization and similarity among *Herbaspirillum* sp. and group I bacteria.

**Figure 3:**
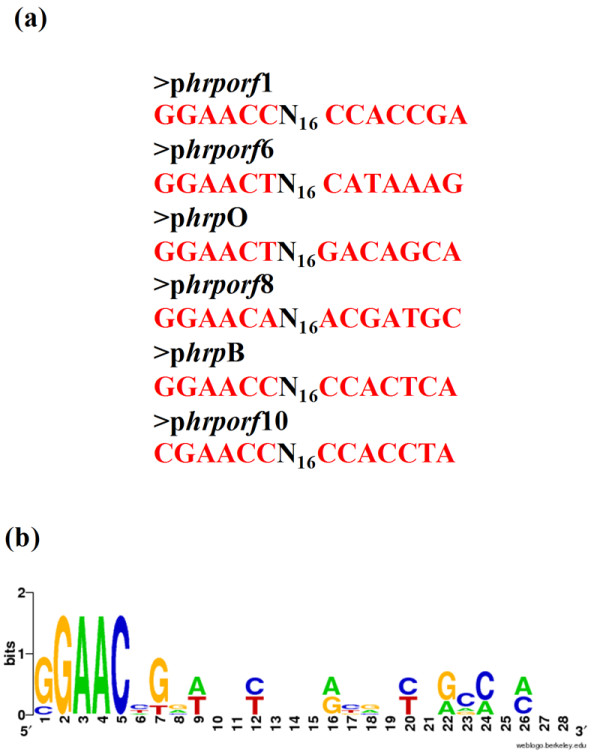
**(a) Putative promoter sequences of the *orf1,orf6, orf8, hrpB *and *orf10 *operons and *hrpO *gene of *H. rubrisubalbicans.*** (**b**) Schematic conserved nucleotide bases found in the promoter regions – *H. rubrisubalbicans* Hrp-box.

Sequences similar to the HrpL-dependent promoter consensus (GGAACC-N15-CCACTCAAT) [26-29] were detected upstream from *orf1*, *orf6*, *hrpO*, *orf8*, *hrpB* and *orf10* (Figure 3a, b). The ORFs from* orf8* to *orf9,* from *hrpB* to *hrpE* and from *orf10* to *hrcC* overlap or are spaced by less than 94 nucleotides apart, suggesting that these three groups of genes are part of three distinct operons. The ORFs from *orf6* to *hrcN* appear to belong to the same operon, although a 114 bp gap is found between *orf6* and *orf7*, but no promoter was found upstream from *orf7*. Likewise, the intergenic regions *orf1orf2* and *orf3orf4* contain 336 bp and 249 bp, respectively, but no promoter sequence was identified. This analysis suggests that *H. rubrisubalbicans hrp/hrc* genes are probably organized in six HrpL-dependent operons.

### *H. rubrisubalbicans hrp* associated genes

Two Hrp associated genes called *hpaB* (JN256204) and *hpaB1* (JN256205) encode general T3SS chaperones, which promote secretion and translocation of multiple effectors proteins [30]. The *hpaB* and *hpaB1* genes are predicted to belong to the TIR chaperone protein family. The *hpaB1* gene was found approximately 12 kb downstream from the *hrcC* gene and it encodes a small acidic chaperone.

### *H. rubrisubalbicans* T3SS effector proteins

Type III secretion systems have been characterized in a variety of plant pathogenic bacteria. The structural proteins of these systems are highly conserved, but the T3SS effector proteins, that play a central role in virulence, are less conserved and difficult to identify.

A BlastX search of the *H. rubrisubalbicans* partial genome sequence (30%) against NCBI-nr database allowed identification of five candidates of *H. rubrisubalbicans* effector proteins HropAN1 (*H. rubrisubalbicans* outer protein) (JN256208), HropAV1 (JN256209), HropF1 (JN256210), Hrop1 (JN256206) and Hrop2 (JN256207) (Table 1). Hrop1 and Hrop2 were also identified as T3SS effectors by the program EffectiveT3 (http://www.effectors.org/) [31]. The genes encoding these proteins are located apart from the *hrp/hrc* genes cluster.

**Table 1:** **Type III-effector proteins of*H. rubrisubalbicans***

**Putative Effector Protein**	**Homology (Gene Bank accession number)**	**Identity/Similarity %**	**Predicted size aa**
HropAV1	type III effector, HopAV1 family [*Ralstonia solanacearum*] (CBJ40351.1)	56/70	784
HropAN1	type III effector Hrp-dependent outer protein [*Burkholderia sp.* Ch1-1] (ZP_06844144.1)	78/86	428
HropF1	XopF1 effector [*Xanthomonas oryzae pv. oryzae* PXO99A] (YP_001911267.1)	31/45	643
Hrop1	type III effector protein (partial sequence central part) [*Ralstonia solanacearum* MolK2] (YP_002252977.1)	25/45	736
Hrop2	leucine-rich-repeat type III effector protein (GALA5) [*Ralstonia solanacearum* PSI07] (YP_003752484.1)	32/46	641

The T3SS putative effectors were identified by BlastX and EffectiveT3 (http://www.effectors.org/) (Arnold *et al.*, 2009).

The proteins HropAN1 (*H. rubrisubalbicans* outer protein), HropAV1 and HropF1 are similar in sequence to HopAN1 (*Burkholderia* sp.), HopAV1 (*Ralstonia solanacearum*) and XopF1 (*Xanthomonas oryzae*), respectively. Hrop1 is homologous to a type III effector protein from *Ralstonia solanacearum* MolK2. Hrop2 belongs to the leucine-rich repeats (LRRs) ribonuclease inhibitor (RI)-like subfamily [32]. The genes encoding HropAV1 and Hrop1 immediately upstream of the *hpaB1* gene, and outside the main T3SS gene cluster.

The *H. rubrisubalbicans* HrpB protein is homologous (identity 27%/similarity 48%) to the *Pseudomonas syringae* HrpB protein that is secreted and contributes to elicitation of the hypersensitive response in *Nicotiana tabacum* and *Nicotiana benthamiana *[33]. This similarity suggests that *H. rubrisubalbicans* HrpB is a candidate for a secreted protein.

*H. rubrisubalbicans hrpE* and *hrcN* genes are essential for the development of mottled stripe disease in sugarcane variety B-4362.

To investigate the contribution of T3SS to the plant-bacterial interaction process we generated the mutants TSN and TSE of *H. rubrisubalbicans* carrying Tn5 insertions in the *hrcN* and *hrpE* genes, respectively. *H. rubrisubalbicans* HrcN protein contains 442 aminoacids and is homologous to T3SS-associated ATPases. The *H. rubrisulbalbicans* HrpE protein contains 202 aminoacids and belongs to the YscL/FliH family of cytoplasmic proteins [34].

**Figure 4:**
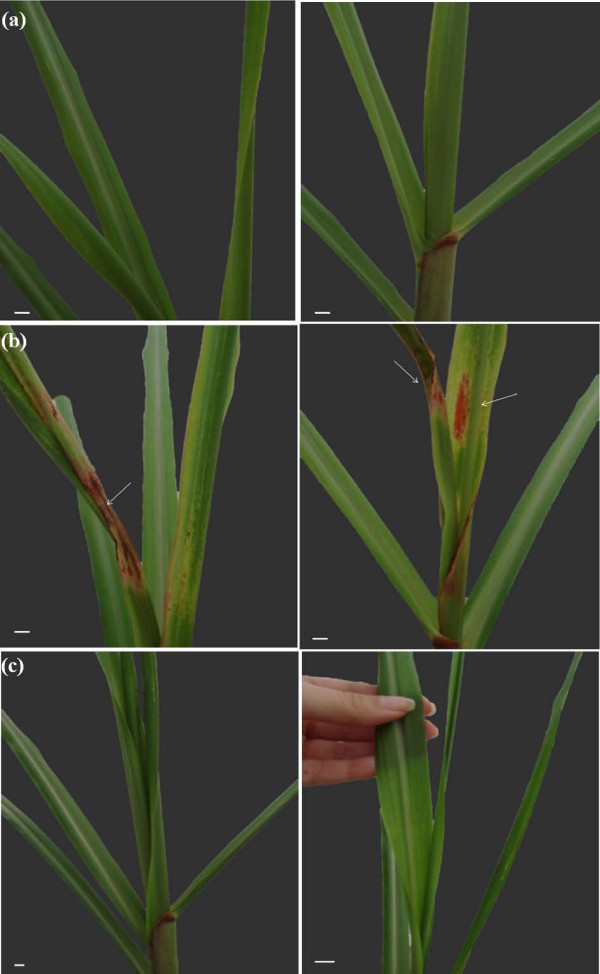
**Inoculation of sugarcane variety B-4362 with wild type and *hrpE *mutant strains of *H. rubrisubalbicans.*** 120 days after germination, 5 sugarcane plants variety B-4362 were inoculated with 10 mM of MgSO_4_ (**a**), *H. rubrisubalbicans* M1 (0.5 – 1.0x10^8^ cells) (**b**) and *H. rubrisubalbicans* TSE (0.5 – 1.0x10^8^ cells) (**c**). The photos were taken 15 days after inoculation (135 days after germination). The arrows indicate bacterial inoculation site and symptoms of the mottled stripe disease (**b**). The scale bars are shown (1 cm).

The wild type M1 and the mutant strains TSN and TSE were inoculated into the susceptible sugarcane variety B-4362. After 15 days, strain M1 caused typical symptoms of mottled stripe disease (mottled background with red stripes and red patches) and well-developed signs of necrosis in leaves invaded by bacteria (Figure 4a). In contrast, the mutants TSN and TSE did not elicit disease symptoms (Figure 4b,c). These results indicate that *hrpE* and *hrcN* gene products are required for the expression of visible symptoms of mottled stripe disease in sugarcane leaves variety B-4362.

**Figure 5:**
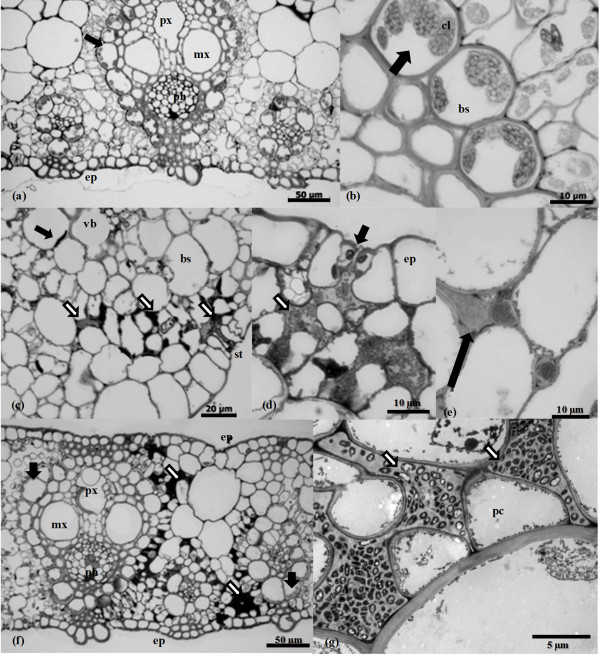
**Light microscopy (LM) and transmission electron microscopy (TEM) of sugarcane leaf blades variety B-4362 inoculated with *H. rubrisubalbicans *M1, TSE and TSN.** (**a**) Transversal section showing the regular tissue organization of a control plant. (ep) epidermis layer, (px) protoxylem, (ph) phloem, (mx) metaxylem, (bu) buliform cells, (arrows) bundle sheath layer with healthy chloroplasts. (**b**) Detailed view of the bundle sheath layer (bs) showing its chloroplasts (cl) with regular shape, distribution and appearance (arrows), and (pc) parenchyma cells. (**c**) Typical pattern of colonization of *H. rubrisubalbicans* strain M1 (wild type) showing tissue system changes associated with extensive colonization of the intercellular spaces of the mesophyll and sub-stomatal cavity (white arrows). Note the chloroplast degradation (black arrow), (vb) vascular bundles, (bs) bundle sheath, (st) stomata. (**d**) Detailed view of the apoplastic colonization by the wild type M1 (white arrow) and beneath the stomata cavity (black arrow). Note the bacteria surrounded by toluidine blue-stained gums. (ep) epidermis. (**e**) Transversal section showing TSE and TSN mutants colonizing the leaf blade. Note the plant gums which restrict the intercellular spreading of the bacterial mutants (black arrow). (**f**) Transversal section of localized areas densely colonized by the mutants (white arrows) showing minor anatomical changes compared with panels (**a**) and (**c**). Note the reduced numbers and size of the bundle sheath chloroplasts (black arrow). (**g**) Transmission electron microscopy of the mutant bacteria colonizing the intercellular spaces of mesophyll cells. See changes in the cytoplasm of the plant host cell in close contact with the bacteria. (pc) parenchyma cells. Three plants of each condition were used for microscopy and the pictures are representative of the three inoculated plants.

Samples of B-4362 leaves inoculated with the strains M1, TSE and TSN were analyzed by light microscopy (LM) and transmission electron microscopy (TEM). Cross sections of the control leaf did not have any visible symptoms and showed the expected anatomical organization for sugarcane foliar blades (Figure 5a). Detailed views of the bundle sheath layer showed chloroplasts of regular shape, distribution and appearance (Figure 5b). In contrast, leaf blades developing symptoms of the mottled stripe disease (inoculated with M1) showed disorganization of the parenchyma tissue characterized by cell wall swelling, hypertrophy and degradation of chloroplasts in both the bundle sheath layer and radial mesophyll cells (Figure 5c). These tissue alterations were associated with extensive colonization of the intercellular spaces of the mesophyll and sub-stomatal cavity by *H. rubrisubalbicans* strain M1 which were surrounded by gum, strongly stained with toluidine blue (Figure 5c,d). In contrast to the wild type (M1), both *H. rubrisubalbicans* mutant strains were not frequently seen in different serial cross sections of the leaf blades. Although all the strains had the same pattern of mesophyll colonization described above (Figure 5c), TSE and TSN mutant strains colonized the leaf blade less extensively. Moreover, more plant gum was present, an indication of an effective host defense which apparently restricted the intercellular spreading of both mutants (Figure 5e). Interestingly, even in areas densely colonized by the mutants, the plant tissue showed only minor anatomic changes, preserving the shape and sizes of the parenchyma cells and vascular bundles (Figure 5e). However, the apoplastic colonization by the mutant strains reduced the numbers and sizes of the bundle sheath chloroplasts and produced changes in the cytoplasm and nuclei of plant host cells in close contact with the bacteria (Figure 5f, g). Taken together these results suggest that although the qualitative pattern of bacterial colonization was not affected, the T3SS is necessary for extensive colonization and to induce plant tissue changes which lead to mottled stripe disease symptoms.

*H. rubrisubalbicans hrpE* and *hrcN* mutant strains do not elicit lesions on *Vigna unguiculata* leaves.

**Figure 6:**
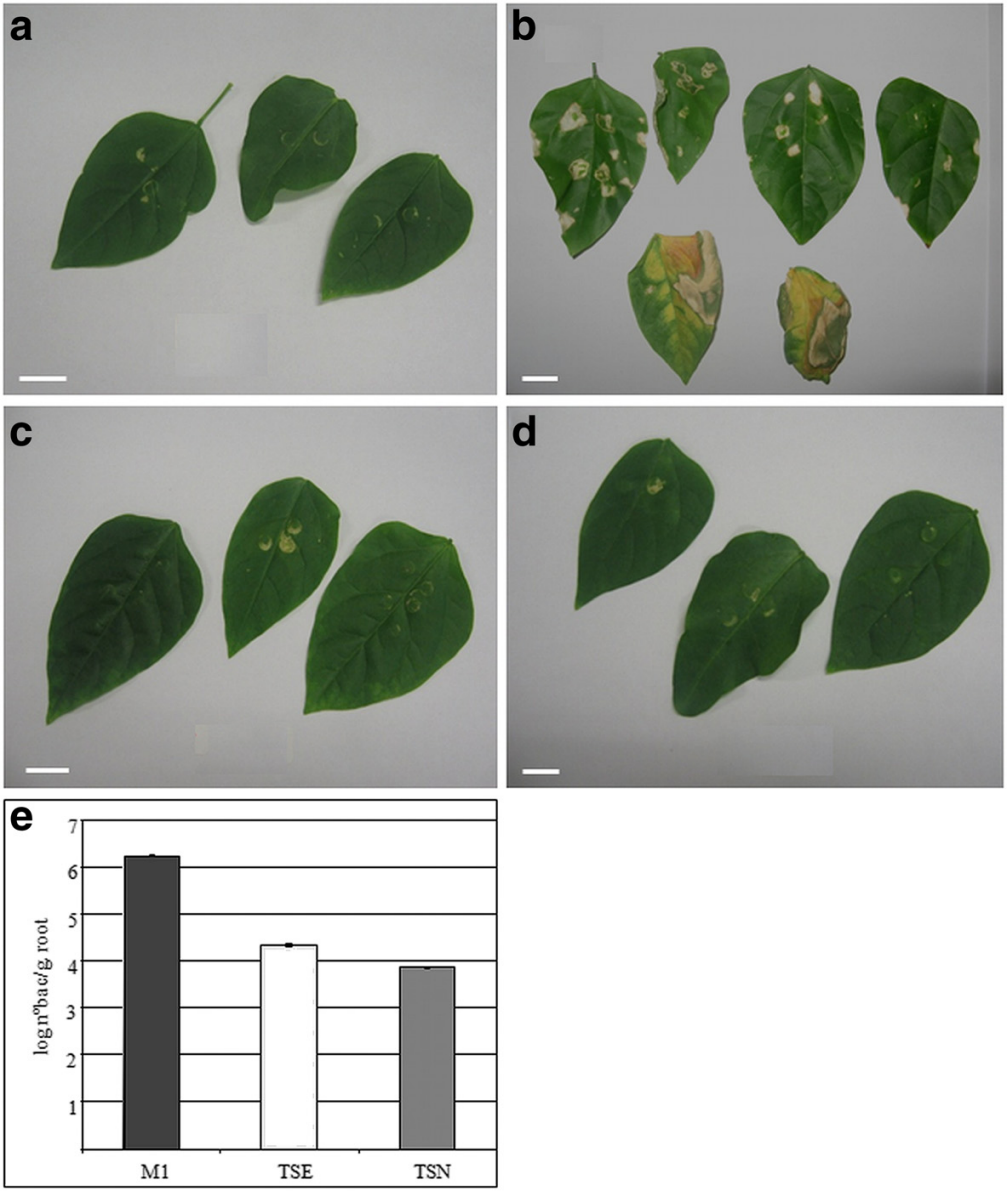
**Inoculation of *Vigna unguiculata *leaves with M1, TSE and TSN strains of *H. rubrisubalbicans *and recovery of bacteria from internal tissue.***V. unguiculata* leaves were infiltrated twenty days after germination; the photos were taken 10 days after infiltration. The scale bars are shown (1 cm). (**a**) Control leaves were infiltrated with 1 mL of MgSO_4_ 10 mM solution. (**b**) Leaves infiltrated with wild type strain M1 (10^8^ cells). (**c**) Leaves infiltrated with 10^8^ cells of the mutant strain TSE. (**d**) Leaves infiltrated with mutant strain TSN (10^8^ cells). (**e**) *V. unguiculata* plants were infiltrated with the indicated strains, and ten days later they were superficially disinfected, macerated, the macerate was diluted and plated. The plates were kept at 30 °C for 24 hours and colonies counted. The experiment contained five plants in each condition and repeated on at least three separate dates. Results are shown as means of Log_10_ (number of bacteria g^-1^ of fresh root). Standard deviation (Student *t*-test, *p* < 0.05).

To study the effect of T3SS genes mutation in another host, *V. unguiculata* leaves were infiltrated with *H. rubrisubalbicans* strains M1, TSE and TSN. Inoculation with *H. rubrisubalbicans* M1 caused lesions on the leaves. The infiltrated zone showed the first sign of tissue collapse after 48 h of infiltration, and within 10 days the zone became necrotic, surrounded by strong chlorotic halos, followed by leaf loss (Figure 6b).

In contrast, infiltration of leaves with *H. rubrisubalbicans* TSE and TSN mutants did not produce lesions (Figure 6c, d). These data suggest that mutation in *hrpE* and *hrcN* genes prevented the TSE and TSN mutant strains from causing disease symptoms on infiltrated leaves.

The leaves of *V. unguiculata* used as controls (Figure 5a) and those inoculated with the wild type M1 and mutant strains TSE and TSN were superficially disinfected, macerated and dilutions were plated. The results show that 10^6^ bacteria/g of fresh weight were recovered from leaves infiltrated with the wild type M1 (Figure 6e), while the number of bacteria from leaves infiltrated with mutant strains TSE and TSN was about 100 times lower (Figure 6e). The decrease in internal colonization is not due to differences in the growth rate since the doubling times of *H. rubrisubalbicans* T3SS mutant strains in NFbHPN medium are identical to the wild type (data not shown). When *Pseudomonas syringae* pv. *tomato* T3SS mutant strains were infiltrated in tomato leaves a reduction in the number of recovered bacteria was also observed [35,36].

These results further support our findings that the genes *hrpE* and *hrcN* are involved in the colonization of *V. unguiculata* by *H. rubrisubalbicans*.

Mutations in *hrpE* and *hrcN* genes reduce the capacity of *H. rubrisulbalbicans* to colonize rice.

**Figure 7:**
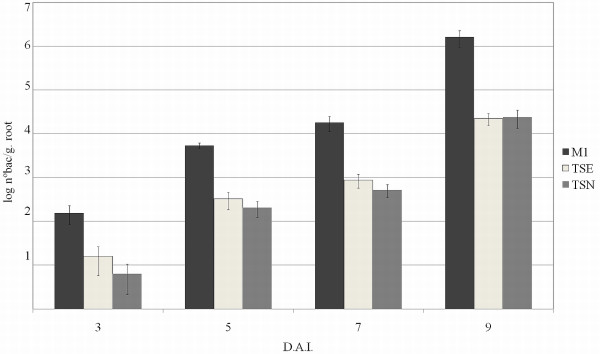
**Internal colonization of *Oryza sativa *roots by *H. rubrisubalbicans*.** The number of endophytic bacteria colonizing internal rice root tissues was determined 3, 5, 7 and 9 days after inoculation (d.a.i.). The plants were superficially disinfected and the roots were cut, homogenized, diluted and plated. The plates were kept at 30°C for 24 hours and colonies counted. Results are shown as means of Log_10_ (number of bacteria. g^-1^ of fresh root) ± standard deviation (Student *t*-test; *P* < 0.05). The experiment contained five different plants for each condition. This experiment was repeated on at least three separate dates.

*H. rubrisubalbicans* has been found in roots and leaves of rice [37] but the interaction was not pathogenic. To investigate if *H. rubrisubalbicans hrcN* and *hrpE* genes are involved in such non-pathogenic endophytic colonization, rice seedlings were inoculated with *H. rubrisubalbicans* strains M1, TSE and TSN five days after germination and the number of endophytic bacteria determined 3, 5, 7 and 9 days after inoculation. No disease symptoms were observed in plants inoculated with any of these bacterial strains. Figure 7 shows that three days after inoculation the number of endophytic wild-type bacteria was 10-fold higher than that of the mutant strains. This difference remained 5 and 7 days after inoculation and increased to 100-fold after nine days. The results indicate that the genes *hrpE* and *hrcN* may also be involved in the endophytic colonization of rice by *H. rubrisubalbicans*.

## Discussion

The type three secretion system of gram-negative plant pathogenic bacteria belonging to the genera *Pseudomonas*, *Ralstonia*, *Xanthomonas* and *Erwinia* is essential for disease development [35]. Bacteria of the genus *Herbaspirillum* endophytically colonize plants of the Poaceae family but can also be found in internal tissues of other plants such as *Phaseolus vulgaris *[38,39] and soybean (*Glycine max*) [40], as well as the tropical species banana and pineapple [41]. Most *Herbaspirillum* species establish neutral or beneficial interaction with plants [42-49]. *H. rubrisubalbicans* can establish non pathogenic beneficial interactions with the *Poaceae* but is also capable of causing disease in some varieties of sugarcane and sorghum [1,2,5]. In this report we show that the T3SS of *H. rubrisubalbicans* is important for establishing pathogenic interactions with sugarcane, lesion formation in *V. unguiculata* leaves as well as endophytic colonization of a rice cultivar and maize.

The gene organization of the *H. rubrisubalbicans hrp/hrc* cluster is identical to that of *H. seropedicae *[25]. The T3SS gene cluster of phytopathogenic bacteria can be divided into two groups based on DNA homology, genetic organization, and regulation pattern [35]. The structural organization of *hrcUhrpXhrcShrcRhrcQ* and *hrpBhrcJhrpDhrpE* genes in the *H. rubrisubalbicans hrp* cluster resembles that of bacteria such as *Pseudomonas syringae*, *Erwinia amylovora*, and *Pantoea stewartii*. *H. rubrisubalbicans* also possesses a *hrpL* gene, a characteristic of bacteria from group I. The HrpL protein, a member of the ECF family of alternative sigma factors, regulates the expression of *hrp* genes in group I [27,50,51]. Interestingly, *H. rubrisubalbicans hrpL* has no σ^54^ promoter sequence, a feature conserved in group I organisms, but contains a gene highly similar to *hrpG*. The HrpG protein is involved in the expression of group II *hrp* genes [52,53]. Upstream from *orf1*, *orf6*, *hrpO*, *orf8*, *hrpB* and *orf10* are conserved sequences that are similar to the *hrp* box sequences which are recognized by HrpL of *P. syringae *[27-29] suggesting the presence of at least six HrpL dependent operons. This is consistent with the observation that *hrp* genes are commonly organized in large gene clusters, consisting of multiple transcriptional units. For instance, *P. syringae* pv. *syringae* and *E. amylovora* contain a 25 Kb cluster with eight transcriptional units [54].

Blast search using the available sequence allowed to identify five candidates for *H. rubrisubalbicans* effector proteins: Hrop1, Hrop2, HropAV1, HropAN1 and HropF1. Only HropAN1 has a counterpart in *H. seropedicae*, the other effector proteins are unique to *H. rubrisubalbicans* and could be involved in the pathogenic phenotype of *H. rubrisubalbicans.*

To determine if the T3SS of *H. rubrisubalbicans* is functional we constructed and characterized *hrcN* and *hrpE* mutants. T3SS-associated ATPases (HrcN proteins) have long been predicted to be the key energizers of the T3SS. The *H. rubrisubalbicans hrcN* mutant failed to cause the mottled stripe disease in sugarcane variety B-4362, demonstrating that the HrcN of *H. rubrisubalbicans* is important for bacterial pathogenicity. Similar results were observed in other plant pathogens, such as *Xanthomonas oryzae* pathovar *oryzae* KACC10859, whose *hrcN* mutant completely lost virulence [55]. *X. campestris pv. vesicatoria* strain 85, whose *hrcN* mutant failed to induce plant reactions in susceptible and resistant pepper plants [56], and a *R. solanacearum hrcN* mutant lost virulence on tomato [57].

The *H. rubrisubalbicans hrpE* mutant also lost the ability to cause disease. This phenotype might be due to direct loss of the function of this gene or could be due to a polar effect on genes downstream from *hrpE*. For example, the gene *hrcC*, which expresses the pore-forming outer membrane protein, is located downstream from *hrpE* and without the pore the external needle effector proteins remain in the cytoplasm or periplasm of the bacteria. This phenotype has been shown for *P. syringae*, where the mutant strain in the *hrpE* gene did not cause a hypersensitive response in plants of *Nicotiana tabacum *[33].

*H. rubrisubalbicans hrcN* and *hrpE* mutants did not elicit lesions on *V. unguiculata* leaves. Thus, our results point to the involvement of the *H. rubrisubalbicans* T3SS in the development of disease symptoms in *V. unguiculata* leaves.

Interestingly, the *H. rubrisubalbicans hrcN* and *hrpE* mutants were less proficient in endophytic colonization of rice and maize, indicating that the T3SS genes have a dual function depending on the host. In susceptible hosts T3SS expression by *H. rubrisubalbicans* leads to the development of disease whereas in symptomless hosts the T3SS is important to avoid the plant response allowing bacterial colonization. Impairment of the T3SS system also produced opposing effects on different plants inoculated with the symbiotic nodulating bacterium *Rhizobium* sp. NGR234 [58]. Some leguminous plants are more effectively nodulated by an *rhcN* (*hrcN* homolog) mutant strain than by the wild type, while others display the opposite behavior. Molecular analysis of this behavior lead to the characterization of effector proteins as being positive, negative or neutral depending on the effect of their removal [59]. Since *H. rubrisubalbicans* strains can stimulate growth of some plants [8] it remains to be determined if the T3SS of such strains can contribute to the beneficial effects.

## Conclusions

Our results showed that a mutation in the *hrpE* and *hrcN* genes lead to a bacterium uncapable to cause the mottled stripe disease in B-4362 sugarcane, indicating that the *H. rubrisubalbicans* T3SS is necessary for the development of the disease. A decrease in rice endophytic colonization was also observed with these mutants, suggesting that in symptomless plants the *H. rubrisubalbicans* T3SS is important for endophytic colonization.

## Methods

### Bacterial strains

The bacterial strains used in this study are listed in Table 2.

**Table 2:** Bacterial strains

**Strains**	**Genotype/phenotype**	**Reference**
*Herbaspirillum rubrisubalbicans* M1	Wild type strain	(BALDANI *et al.*, 1996)
*Herbaspirillum rubrisubalbicans* TSE	M1 *hrpE*^-^ EZ::Tn5^TM^ < TET1>, Tc^R^, Km^R^	This work
*Herbaspirillum rubrisubalbicans* TSN	M1 *hrcN*^-^ EZ::Tn5^TM^ < TET1>, Tc^R^	This work
*Escherichia coli* TOP10	F^-^*mcrA* Δ(*mcrr-hsdRMS-mcr*BC) φ80*lacZ*ΔM15 Δ*lac*ZX74 *doe*R *rec*A1 *endA1 ara*Δ139 Δ(*ara, leu*) 7697 *gal*U *galK* λ^-^*rpsL nupG* λ^-^	INVITROGEN

### Media and growth conditions

*Escherichia coli* was grown at 37°C in LB medium [60]. Strains of *H. rubrisubalbicans* were grown at 30°C in NFbHPN-malate [61]. Antibiotics were used at the following concentrations: tetracycline 10 μg ml^−1^, ampicillin 250 μg ml^−1^, chloramphenicol 30 μg ml^−1^, and kanamycin 50 μg ml^−1^ for *E. coli* strains and 100 μg ml^−1^ for *H. rubrisubalbicans* strains.

### *H. rubrisubalbicans hrp/hrc* genes sequencing

Partial sequencing of the *H. rubrisubalbicans* M1 genome (Monteiro *et al.*, unpublished) revealed the presence of T3SS genes. *hrp/hrc* gene specific primers were designed to amplify and sequence gaps to obtain the whole sequence of the T3SS gene cluster. DNA sequence reactions were analyzed with an ABI PRISM 377 automatic DNA sequencer (Applied Biosystems, California, USA).

### Phylogenetic analyses

Phylogenetic and molecular evolutionary analyses were conducted using MEGA version 5 [62]. DNA sequences were retrieved from GenBank database, translated to amino acids sequences and aligned using Muscle [63] with the following option differing from default: gap opening −12, gap extension −1, and hydrophobicity multiplier 1. Redundancy for sequences showing less than 0.1 p-distances were eliminated to avoid any bias, then the remaining sequences were realigned. Aligned amino acids sequences were converted back to nucleotide sequences and used to perform phylogenetic analysis. Alignment of protein sequences allow the use of substitution matrix and avoid gap insertion within codons. The Maximum Likelihood (ML) method was used to test the evolutionary models giving best results with Tamura 3-parameters, with gamma-distribute rates and invariant sites model. The selected model was used to build a phylogenetic tree using the ML method with 1,000 bootstrap replicates. Option for partial deletion with site coverage of 95% and a phylogenetic tree built using Neighbor-Joining (NJ) method with Kimura 2-parameter calculated distances and 10,000 bootstrap replicates was used as a start tree for all ML analysis.

Edition in phylogenetic tree was made using FigTree version 1.3.1 (http://tree.bio.ed.ac.uk/).

### Plant assays

Bacterial cultures of *H. rubrisubalbicans* M1 were grown in NFbHPN-malate [61] medium at 30°C for 18 h with shaking (120 rpm).

Sugarcane variety B-4362 cuttings were obtained from the Program for Genetic Improvement of Sugarcane - CECA/UFAL. These were surface disinfected by treatment with Karate 0.1% and Derosal 0.01% for 2 minutes and heat treatment (immersion in water at 52°C for 30 minutes). Sugarcane inoculation was performed as described [1]. 120 days after germination the stalks of sugarcane were inoculated by injecting with a hypodermic syringe 0.5 to 1 mL of cell suspension in 10 mM MgSO_4_ (10^8^ cfu mL^−1^) into the foliar cartridge 2 to 3 cm below the first leaf. After inoculation the leaves were pruned halfway, and the plant was wrapped with a plastic bag to maintain a high humidity environment. Sugarcane inoculated with *H. rubrisubalbicans* was visually inspected for mottled stripe disease 15 days after inoculation. *Vigna unguiculata* cultivar *Red Caloona* seeds were sterilized with 97% sulfuric acid for 10 minutes, followed by four washes with sterilized water [64]. The seeds were germinated in pots containing vermiculite and BD nutrient solution [65] and cultivated at 30°C with a 16 h light period.

Bacterial suspensions (10^8^ cfu mL^−1^) in 10 mM MgSO_4_ were infiltrated into the abaxial leaf surface of twenty days old *V. unguiculata* using a syringe without a needle. The plants were kept in a greenhouse at 30°C, illuminated by sunlight and watered every three days. To determine the number of endophytic bacteria, ten days after *H. rubrisubalbicans* infiltration, leaves were superficially disinfected with 70% ethanol for five minutes, washed with sterilized water and homogenized with a sterile pestle and mortar in 1 mL of sterile PBS. Leaf extracts were serially diluted and used to determine the number of bacteria colonizing internal plant tissues by plating on NFbHPN-malate.

*Oryza sativa* L. ssp. *japonica* seeds (variety BRS Formosa) were surface-sterilized with ethanol 70% for 1 min then shaken in 6% hypochlorite and 0.02% tween 20 for 30 min at 30°C, and washed three times with sterile water. The seeds were germinated in Petri dishes containing 1% agar at 25°C for 120 h. Plants were grown in an incubator at 25°C with a 16 h light period and 60% humidity. Thirty seedlings were inoculated five days after germination with 30 mL of *H. rubrisubalbicans* strains suspension (10^8^ cfu mL^−1^) by immersion for 15 minutes. The seedlings were transferred to glass tubes containing 20 mL of Hoagland medium [66] with 0.2% agar and maintained at 25°C, 16 h light period. The roots were cut 3, 5, 7 and 9 days after inoculation, weighed before surface sterilization by a 2 minutes wash with 1% sodium hypochlorite containing 0.01% tween-20, followed by 2 minutes in 70% ethanol, and four washes with sterile distilled water. The samples were then homogenized using a sterile pestle and mortar, and the root extracts diluted in 1 mL of sterile PBS. The number of bacteria colonizing internal plant tissues was determined by plating several dilutions of the extracts on NFbHPN-malate plates. The results reported here represent the average of at least five independent experiments.

### Recombinant DNA techniques

Standard procedures were performed for plasmid DNA extraction, restriction enzyme reactions, cloning and bacterial transformations [60 or according to the manufactures recommendations].

### Construction of *H. rubrisubalbicans hrpE* and *hrcN* mutant strains

The genes *hrpE* and *hrcN* of *H. rubrisubalbicans* in plasmids HR02-MF-00-000-009-C05.km and HR02-MF-00-000-053-F11.km (Monteiro and Petruzziello, unpublished) were disrupted by the transposon EZ:: Tn5^TM^ < TET1 > (Epicentre) that confers resistance to tetracycline. The mutant suicide plasmids were electroporated into the wild type *H. rubrisubalbicans* strain M1. Recombinant cells were selected for tetracycline resistance and screened for the loss of kanamycin resistance (vector marker). Southern blot analyses of EcoRI digested genomic DNA were used to confirm the presence of the tetracycline transposon in *hrpE* and *hrcN.* (data not shown). The selected mutant strains, named TSE and TSN, contained transposon insertions into *hrpE* and *hrcN,* respectively.

### Light and transmission electron microscopy

Leaves were taken at 21 days after inoculation, washed twice in phosphate buffer (50 mM, pH 7.0) and fixed in 2.5% (v/v) glutaraldehyde (in 50 mM phosphate buffer, pH 7.0). Leaf sections were prepared for light and transmission electron microscopy according to James *et al. *[67].

## Authors’ contributions

Conceived and designed the work: FOP, RAM and EMS. Performed the experiments: MAS, EB, RW, HF, FLO and VAB. Performed assembly, annotation, and bioinformatics analyses: MAS, EB, RW, LMC, VAW, HF, EMS, RAM, HMFM, LPF, MHPF, FMP, LFPP, LGEC. Wrote the manuscript: RAM, EMS, MGY and MAS. Prepared figures: LMC, RAM, EB and MAS. All authors read and approved the final manuscript.

## Supplementary Material

Additional file 1 **Table S1.** Aminoacids sequence homology between Hrp/Hrc proteins of *H. rubrisubalbicans* and *H. seropedicae*. These data show the identity and similarity between the T3SS proteins from *H. rubrisubalbicans* and *H. seropedicae*. Click here for file
