# Supplementary material for: The type III secretion system is necessary for the development of a pathogenic and endophytic interaction between Herbaspirillum rubrisubalbicans and Poaceae
Source: BMC Microbiol. 2012 Jun 6;12:98. doi: 10.1186/1471-2180-12-98 (PMC3487950; doi:10.1186/1471-2180-12-98)
Supplement: Additional file 1 — Table S1. Aminoacids sequence homology between Hrp/Hrc proteins of H. rubrisubalbicans and H. seropedicae. These data show the identity and similarity between the T3SS proteins from H. rubrisubalbicans and H. seropedicae. [file 1471-2180-12-98-S1.doc]

Supporting Information – Table S1 - Aminoacids sequence homology between Hrp/Hrc proteins of *H. rubrisubalbicans* and *H. seropedicae*

| Protein | Homolog (Gene Bank accession number) | Identity/Positives | Predicted size aa  *H.rubrisubalbicans*/*H.seropedicae* |
| --- | --- | --- | --- |
| HrpL | [ref|YP_003774207.1|](http://www.ncbi.nlm.nih.gov/protein/300310115?report=genbank&log$=protalign&blast_rank=1&RID=FFR9Z9NB01S) RNA polymerase sigma-24 factor protein [*Herbaspirillum seropedicae* SmR1] | 57.1% /67.2% | 202/244 |
| Hrorf1 | ref|YP_003774208.1| hypothetical protein Hsero_0781 [*Herbaspirillum seropedicae* SmR1] | 20.2%/30% | 277/349 |
| Hrorf2 | ref|YP_003774209.1| hypothetical protein Hsero_0782 [*Herbaspirillum seropedicae* SmR1] | 28.2%/42.5% | 171/127 |
| Hrorf3 | ref|YP_003774210.1| hypothetical protein Hsero_0783 [*Herbaspirillum seropedicae* SmR1] | 37.9%/57.8% | 101/108 |
| Hrorf4 | ref|YP_003774211.1| hypothetical protein Hsero_0784 [*Herbaspirillum seropedicae* SmR1] | 39.9%/56.9% | 145/151 |
| Hrorf5 | ref|YP_003774212.1| hypothetical protein Hsero_0785 [*Herbaspirillum seropedicae* SmR1] | 46.6%/63.1% | 94/99 |
| HrcV | [ref|YP_003774213.1|](http://www.ncbi.nlm.nih.gov/protein/300310121?report=genbank&log$=protalign&blast_rank=1&RID=FFRVE0X901N) type III secretion HrcV transmembrane protein [*Herbaspirillum seropedicae* SmR1] | 83.5%/ 90.9% | 689/695 |
| Hrorf6 | ref|YP_003774214.1| hypothetical protein Hsero_0787 [*Herbaspirillum seropedicae* SmR1] | 10.9%/16.5% | 168/299 |
| Hrorf7 | ref|YP_003774215.1| hypothetical protein Hsero_0788 [Herbaspirillum seropedicae SmR1] | 43.6%/64% | 141/170 |
| HrpQ | [ref|YP_003774216.1|](http://www.ncbi.nlm.nih.gov/protein/300310124?report=genbank&log$=protalign&blast_rank=1&RID=FFS211YD01S) type III secretion HrpQ protein [*Herbaspirillum seropedicae* SmR1] | 48.6%/62.2% | 345/344 |
| HrcN | [ref|YP_003774217.1|](http://www.ncbi.nlm.nih.gov/protein/300310125?report=genbank&log$=protalign&blast_rank=1&RID=FFS7330A01N) type III secretion ATP synthase [*Herbaspirillum seropedicae* SmR1 | 83.3%/89.5% | 459/460 |
| HrpO | [ef|YP_003774218.1|](http://www.ncbi.nlm.nih.gov/protein/300310126?report=genbank&log$=protalign&blast_rank=1&RID=FFSDHRTE01S) hypothetical protein Hsero_0791 [*Herbaspirillum seropedicae* SmR1] | 46.5%/58.8% | 157/170 |
| Hrorf8 | ref|YP_003774219.1| hypothetical protein Hsero_0792 [Herbaspirillum seropedicae SmR1] | 31%//43.2% | 202/218 |
| HrcQ | [ref|YP_003774220.1|](http://www.ncbi.nlm.nih.gov/protein/300310128?report=genbank&log$=protalign&blast_rank=1&RID=FFSJYDUD01S) type III secretion system protein [*Herbaspirillum seropedicae* SmR1] | 35.3%/45.5% | 365/400 |
| HrcR | [ref|YP_003774221.1|](http://www.ncbi.nlm.nih.gov/protein/300310129?report=genbank&log$=protalign&blast_rank=1&RID=FFSRBUHV01S) type III secretion HrcR transmembrane protein [*Herbaspirillum seropedicae* SmR1] | 80.9%/92.7% | 219/220 |
| HrcS | [ref|YP_003774222.1|](http://www.ncbi.nlm.nih.gov/protein/300310130?report=genbank&log$=protalign&blast_rank=1&RID=FFSUZKZB01N) type III secretion HrcS protein [*Herbaspirillum seropedicae* SmR1] | 86.2%/96.6% | 87/87 |
| HrcT | [ref|YP_003774223.1|](http://www.ncbi.nlm.nih.gov/protein/300310130?report=genbank&log$=protalign&blast_rank=1&RID=FFSUZKZB01N) type III secretion HpX protein [*Herbaspirillum seropedicae* SmR1] | 76.9%/90.2% | 264/262 |
| HrcU | [ref|YP_003774224.1|](http://www.ncbi.nlm.nih.gov/protein/300310132?report=genbank&log$=protalign&blast_rank=1&RID=FFT3SHW301S) type III secretion HrcU transmembrane protein [*Herbaspirillum seropedicae* SmR1] | 64.7%/77.1% | 366/371 |
| Hrorf9 | ref|YP_003774225.1| hypothetical protein Hsero_0798 [*Herbaspirillum seropedicae* SmR1] | 26.6%/35.8% | 184/176 |
| HrpB | ref|YP_003774226.1| type III secretion HrpB protein [*Herbaspirillum seropedicae* SmR1] | 52.9%/72.5% | 133/134 |
| HrcJ | [ref|YP_003774227.1|](http://www.ncbi.nlm.nih.gov/protein/300310135?report=genbank&log$=protalign&blast_rank=1&RID=FFT8AE4801S) type III secretion HrcJ lipoprotein [*Herbaspirillum seropedicae* SmR1] | 65.1%/76.4% | 282/292 |
| HrpD | ref|YP_003774228.1| HrpD-like protein [*Herbaspirillum seropedicae* SmR1] | 36%/47.4% | 208/215 |
| HrpE | [ref|YP_003774229.1|](http://www.ncbi.nlm.nih.gov/protein/300310137?report=genbank&log$=protalign&blast_rank=1&RID=FFTCYJ7301S) type III secretion HrpE protein [*Herbaspirillum seropedicae* SmR1] | 38.3%/53.8% | 202/233 |
| Hrorf10 | ref|YP_003774230.1| hypothetical protein Hsero_0803 [*Herbaspirillum seropedicae* SmR1] | 64.9%/83.1% | 75/77 |
| Hrorf11 | ref|YP_003774231.1| hypothetical protein Hsero_0804 [*Herbaspirillum seropedicae* SmR1] | 45.3%/60.4% | 138/137 |
| HrcC | [ref|YP_003774232.1|](http://www.ncbi.nlm.nih.gov/protein/300310140?report=genbank&log$=protalign&blast_rank=1&RID=FFTH60V901N) type II/III HrcC transmembrane protein [*Herbaspirillum seropedicae* SmR1] | 64.2%/75.4% | 701/693 |

A pairwise sequence alignment was performed for each pair of genes in *H. rubrisubalbicans* and its homologous counterpart in *H. seropedicae*. The global alignment was carried out using Needle tool, available through EMBL-EBI web site (www.ebi.ac.uk), using the default parameters.
